# Epidemiology and pathology of avian malaria in penguins undergoing rehabilitation in Brazil

**DOI:** 10.1186/s13567-015-0160-9

**Published:** 2015-03-13

**Authors:** Ralph Eric Thijl Vanstreels, Rodolfo Pinho da Silva-Filho, Cristiane Kiyomi Miyaji Kolesnikovas, Renata Cristina Campos Bhering, Valeria Ruoppolo, Sabrina Epiphanio, Marcos Amaku, Francisco Carlos Ferreira Junior, Érika Martins Braga, José Luiz Catão-Dias

**Affiliations:** Departamento de Patologia, Faculdade de Medicina Veterinária e Zootecnia, Laboratório de Patologia Comparada de Animais Selvagens, Universidade de São Paulo, Avenida Orlando Marques de Paiva 87, São Paulo, SP 05088–000, Brazil; Centro de Recuperação de Animais Marinhos, Museu Oceanográfico Professor Eliézer de Carvalho Rios, Universidade Federal do Rio Grande, Rua Capitão Heitor Perdigão 10, Rio Grande, RS 92200-580 Brazil; Associação R3 Animal. Rodovia João Gualberto Soares, Entrada do Parque Estadual do Rio Vermelho, Barra da Lagoa, Florianópolis, SC 88061-500 Brazil; Instituto de Pesquisa e Reabilitação de Animais Marinhos. Rodovia BR 262, Instituto Estadual de Meio Ambiente e Recursos Hídricos, Jardim América, Cariacica, ES 29140-130 Brazil; International Fund for Animal Welfare, 290 Summer Street, Yarmouth Port, MA 02675 USA; Departamento de Análises Clínicas e Toxicológicas, Faculdade de Ciências Farmacêuticas, Universidade de São Paulo, Avenida Professor Lineu Prestes 580, Butantã, São Paulo, SP 05508-000 Brazil; Departamento de Medicina Veterinária Preventiva e Saúde Animal, Laboratório de Epidemiologia e Bioestatística, Faculdade de Medicina Veterinária e Zootecnia, Universidade de São Paulo, Avenida Professor Orlando Marques de Paiva 87, São Paulo, SP 05088-000 Brazil; Departamento de Parasitologia, Instituto de Ciências Biológicas, Universidade Federal de Minas Gerais. Caixa Postal 486, Avenida Antônio Carlos 6627, Pampulha, Belo Horizonte, MG 31270-901 Brazil

## Abstract

Seabird rehabilitation is a valuable strategy to mitigate the impacts of oil pollution and other anthropogenic factors, and can significantly contribute to the conservation of penguins. However, infectious diseases such as avian malaria (*Plasmodium* spp.) can hamper the success of rehabilitation efforts. We combined morphological and molecular diagnostic methods to investigate the epidemiology and pathology of *Plasmodium* in Magellanic penguins (*Spheniscus magellanicus*) at rehabilitation centers along 2500 km of the coastline of Brazil. True prevalence of malarial parasites was estimated between 6.6% and 13.5%. We identified five species, three of which had not been described infecting penguins (*P. cathemerium*, *P. nucleophilum*, *P. unalis*); an additional five distinct *Plasmodium* lineages were also distinguished, and albeit unidentified these clearly correspond to species that also have not yet been reported in penguins. Our results indicate that the diversity of plasmodia that may infect these birds is greater than previously recognised. Considering the well-defined seasonality observed in this study, it is clear that rehabilitation centers could benefit by narrowing their preventative efforts on penguins maintained or admitted during the Austral spring-summer, particularly by preventing mosquitoes from coming into contact with penguins.

**Electronic supplementary material:**

The online version of this article (doi:10.1186/s13567-015-0160-9) contains supplementary material, which is available to authorized users.

## Introduction

Avian malaria is a disease caused by mosquito-transmitted protozoans of the genus *Plasmodium*, and more than 60 species are known to infect birds [1]. *Plasmodium* infections tend to be asymptomatic or pose only minor impact on fitness and survival of most species of birds, however a few avian groups are considered highly susceptible and may develop severe disease when exposed to these parasites [2]. Penguins (Sphenisciformes) are one such group, and avian malaria is an important infectious diseases for these birds, especially in a captive environment [3,4]. Nearly all cases of avian malaria in penguins have been attributed to *P. relictum* and/or *P. elongatum* [3,5,6], with only isolated reports of infection by *P. juxtanucleare* [7] and *P. tejerai* [8].

Magellanic penguins (*Spheniscus magellanicus*) breed along the coast of Argentina, Chile and the Malvinas-Falkland Islands [9], and are susceptible to the disease. There are reports of *Plasmodium* sp infections in Magellanic penguins (MPs) at zoos in the United States [10], South Korea [11] and Brazil [12], and at rehabilitation centers in Chile [13] and Brazil [8,14]. In each of these reports, avian malaria has led to rapid outbreaks with high morbidity and mortality of MPs. In contrast, none of the studies examining blood or tissue samples of wild MPs have found evidence of *Plasmodium* sp or other blood parasites, nor have studies on sympatric populations of other penguin species [5,6].

The fact that there are reports of *Plasmodium* sp in MPs in captivity and undergoing rehabilitation but not in the wild suggests the MPs are *Plasmodium*-free when admitted to rehabilitation centers, and acquire the infection during their time in these facilities. We examine this hypothesis by conducting a broad investigation for *Plasmodium* spp. in MPs at rehabilitation centers along the coast of Brazil, determining spatial and temporal distribution of malarial infections, mortality and lineages.

## Materials and methods

### Study locations and data collection

**Figure 1:**
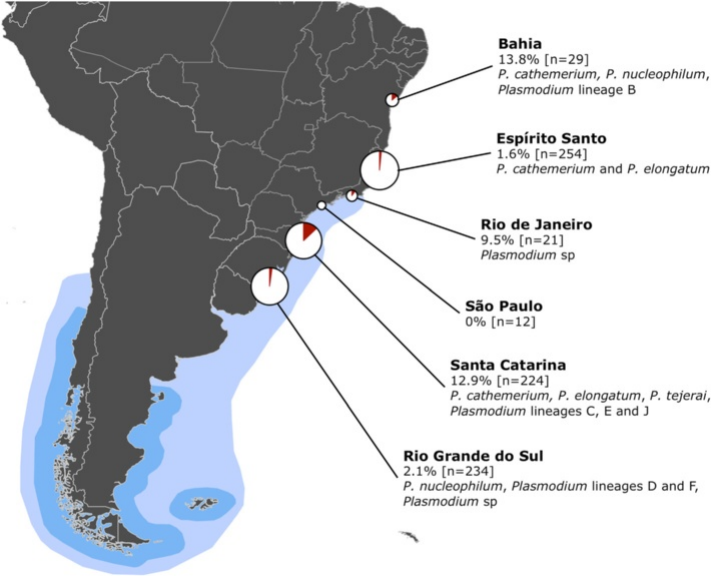
**Geographic distribution of the sampling effort**, **detection and lineages of** ***Plasmodium*** **spp.** Pie charts represent sampling effort (size) and percentage of positive results (red fraction). Blue areas represent the wintering (light blue) and breeding (darker blue) distribution of Magellanic penguins [9].

We studied MPs received for rehabilitation at five organizations along the coast of Brazil (Figure 1): CRAM-FURG (Rio Grande, Rio Grande do Sul - 32°01’34”S 52°06’21”W), CETAS Florianópolis (Florianópolis, Santa Catarina - 27°35’51”S 48°26’20”W), FUNDAMAR (São Sebastião, São Paulo - 23°49’21”S 45°24’53”W), CETAS Unimonte (São Vicente, São Paulo - 23°56’50”S 46°23’39”W), and IPRAM (Cariacica, Espírito Santo - 20°19’54”S 40°21’38”W). Each organization receives penguins rescued along the coastline of their state and neighboring states; additionally, CRAM-FURG also receives then releases penguins that were rehabilitated at other states (Bahia, Espírito Santo, Rio de Janeiro). Considering this dynamic, each penguin was assigned a “location” (Bahia, Espírito Santo, Rio de Janeiro, São Paulo, Santa Catarina, Rio Grande do Sul) based on the facility in which they were subjected to rehabilitation (not rescue nor release location).

Samples were collected in different time periods at each location (Table 1). Samples were collected systematically from January 2009 to December 2012 at Rio Grande do Sul and from March 2009 to February 2013 at Santa Catarina; in these periods, MPs were evenly sampled without bias towards clinically ill or healthy individuals and over an extended time period (“systematic sampling”). Other samples were collected from other facilities over a short period and/or from penguins showing clinical or necropy findings suggestive of infectious disease (“opportunistic sampling”).

**Table 1:** **Sample sizes examined using different diagnostic tests to screen for** ***Plasmodium*** **sp infections**

**Latitude**	**Study site** **(time period)**	**Blood smears** **+** **PCR**	**PCR**	**Blood smears**	**Histopathology**	**Total**
13°00’S	Bahia (1999–2008)^‡^	7 (1)	-	7 (1)	-	8 (2)
13°00’S	Bahia (Jun2009-Dec2012)^‡^	21 (2)	-	-	-	21 (2)
20°20’S	Espírito Santo (1999–2008)^‡^	2	-	17 (1)	-	19 (1)
20°20’S	Espírito Santo (Sep2012)	86	-	111	-	197
20°20’S	Espírito Santo (Sep2012-Feb2013)	18 (2)	20 (1)	-	-	38 (3)
22°50’S	Rio de Janeiro (1999–2008)^‡^	6	1 (1)	5	1 (1)	13 (2)
22°50’S	Rio de Janeiro (Jan2009-Dec2012)^‡^	2	-	6	-	8
23°58’S	São Paulo (Aug2010-Sep2010)	1	11	-	-	12
27°36’S	Santa Catarina (Mar2009-Feb2013)^†^	106 (19)	81 (8)	37 (2)	-	224 (29)
32°02’S	Rio Grande do Sul (1999–2008)	-	-	11	2 (2)	13 (2)
32°02’S	Rio Grande do Sul (Jan2009-Dec2012)^†^	192 (3)	8	21	-	221 (3)
**Total**	Systematic sample collection	298 (22)	89 (8)	58 (2)	-	445 (32)
	Opportunistic sample collection	143 (5)	32 (2)	151 (2)	3 (3)	329 (12)
	Grand total	441 (27)	121 (10)	209 (4)	3 (3)	774 (44)

Values within parenthesis indicate the number of positive samples. “†” indicates that sample collection was systematic, i.e. was not conducted in a manner that could favor sick or healthy individuals. “‡” indicates samples collected upon admission to CRAM-FURG from penguins that had been rehabilitated at other facilities.

Samples and biological information collected within 0–3 days from intake to the facility were considered “intake”, whereas those collected 0–7 days before death or release were considered “exit”. Each individual was categorised in relation to their “age group” on intake (juvenile, adult), “oiling” on intake (oiled, not oiled), “survival” during rehabilitation (survivor, deceased) and “diagnosis” (positive, negative). Rehabilitation records were used to determine “warm season period”, i.e. the number of days spent in the facility (from intake to death or release) that occurred within the period 01 October to 31 March.

### Study design

Screening for *Plasmodium* combined one or more of the following diagnostic methods: thin blood smears (TBS), nested polymerase chain reaction (PCR) and histopathology (HP).

*Plasmodium* screening was divided in two stages. The following criteria were adopted in the first stage: (a) all TBS collected at all study sites were examined; (b) for “survivor” penguins, the “exit” blood sample was tested with PCR; (c) for “deceased” penguins from which frozen tissue samples had been collected at necropsy, these tissues were tested with PCR; (d) for “deceased” penguins from which frozen tissue sampled were not collected at necropsy, the last blood sample collected before death was tested with PCR; (e) for “deceased” penguins from which neither blood nor frozen tissue samples were available, tissue samples in formalin were examined by HP. At this stage, blood smear examination was blind to PCR results and vice-versa.

The second stage of *Plasmodium* screening used the criteria: (a) if a penguin obtained a positive or inconclusive result for one or more samples in the first step, all samples available from that individual were tested with PCR; (b) if a penguin obtained a positive result, all samples from other individuals that had been at the same facility at the same date or three weeks before or following to the date of collection of the positive sample were tested with PCR. Because distinct sets of samples were available for each individual, different combinations of diagnostic tests were used to detect *Plasmodium* among study sites (Table 1).

Finally, individuals with positive results in the previous steps were further tested: (a) positive samples were subjected to sequencing of the *cyt*-*b* gene; (b) histopathology of all available tissue samples were evaluated to determine microscopic lesions and determine the occurrence of exoerythrocytic meronts; (c) all available blood smears were used to characterize parasite morphology.

### Sample collection, hematology and pathology

MPs at the study facilities are subjected to standardized rehabilitation protocols under the supervision of veterinarians [15]. At all facilities involved in the study, external and internal enclosures in which rehabilitation is conducted were not entirely protected against mosquitoes and were within 500 metres from bodies of freshwater and/or fragments of Atlantic forest. Penguins were physically restrained and blood samples were collected from the jugular or metatarsal veins. Body mass was determined with a scale with ± 5 g precision; when these data were collected upon intake, it was referred to as “intake mass”. Thin blood smears and heparin capillaries were prepared immediately after blood collection; the remaining blood was stored in tubes with heparin or without anticoagulants, then frozen. In some cases, hematocrit was determined through centrifugation in heparin capillaries at 16 000 *g* for 5 min; total plasma protein was determined with a clinical refractometer.

Blood smears were dried at room temperature, fixed with absolute methanol, stained with Giemsa or Wright-Rosenfeld stain, and examined under 1000× magnification (field of view area = 0.126 mm^2^). A minimum 150 fields (~30 000 erythrocytes) were examined during the first stage of screening and an additional 250 fields (~50 000 erythrocytes) were examined during the second stage. Blood parasites were morphologically characterized [16] and quantified with the assistance of digital image analysis to count 10 000 erythrocytes [17]; parasite forms were differentiated into four categories (trophozoite, meront, microgametocyte and macrogametocyte).

Whenever possible, penguins that died during rehabilitation were refrigerated and examined within 12 to 24 h after death; when this was not possible, carcasses were frozen for later examination. Gross lesions were photographed and noted, and samples of organs and tissues were fixed in 10% buffered formalin. Formalin-fixed tissues were embedded in paraffin and sections of 3 or 5 μm were obtained, stained with hematoxylin-eosin and examined under light microscopy.

### Molecular biology and phylogenetic analysis

Frozen samples of blood and tissues (lung, spleen or liver) were used for molecular analyses. DNA extraction was conducted using the DNEasy Blood and Tissue Kit (#69506, Qiagen) and was verified and quantified through UV spectrophotometry (Nanodrop 2000, Thermo Fisher Scientific). We used a nested polymerase chain reaction targeting the mitochondrial cytochrome b (*cyt*-*b*) gene of *Haemoproteus* and *Plasmodium* [18] with 3 ng/μL of sample DNA, 0.6 μM of each primer, and GoTaq Green Master Mix 2x (M7122, Promega). Blood samples from chicken experimentally infected with *Plasmodium gallinaceum* and samples from chickens raised in arthropod-free environments were used as positive and negative controls, respectively. Gel electrophoresis was conducted to visualize amplification products, using 2% agarose gel, SYBR Safe (S33102, Invitrogen), and a high-resolution imaging system (Gel Doc EZ System 170–8270, Bio-Rad). PCR amplification products of positive samples were purified with Polyethylene Glycol 8000. Bi-directional sequencing with dye-terminator fluorescent labeling was performed through automated sequencing (ABI Prism 3100, Applied Biosystems); forward and reversed chromatograms were edited and consensus sequences were deposited in GenBank (Additional file 1).

Phylogenetic relationships among *Plasmodium* lineages identified in this study and related hemosporidian parasites were inferred by using sequences from reference lineages from the MalAvi database [19], for which species was identified based on studies using morphological evidence, as well as penguin-infecting *Plasmodium* lineages from published studies (Additional file 1). Sequences were aligned using ClustalW [20] as implemented in MEGA 5.2.2 [21]. A Bayesian phylogenetic tree for the parasite sequences was produced using MrBayes 3.2.2 [22] with the GTR + I + G model of nucleotide evolution, as recommended by ModelTest [23]. We ran two Markov chains simultaneously for 5 million generations that were sampled every 1000 generations. The first 1250 trees (25%) were discarded as a burn-in step and the remaining trees were used to calculate the posterior probabilities.

### Statistical analysis

“Apparent prevalence” was defined as the number of positive individuals divided by the number of individuals tested. “Survival ratio” was defined as the number of “survivor” individuals divided by the total number of individuals in a given data subset. “Lethality” was defined as the number of “deceased” individuals divided by the total number of individuals infected by a given *Plasmodium* lineage.

Chi-Square test was used to compare diagnosis (dependent variable – DV) among laboratory methods (independent variable – IV) (TBS + PCR, PCR, TBS; histopathology was not included due to small sample size).

All subsequent analyses in this subsection were restricted to data obtained from systematically sampled and PCR-tested individuals. True prevalence was estimated from apparent prevalence using Blaker’s 95% confidence interval [24] assuming a 80% sensitivity [25,26] and 100% specificity.

Mann–Whitney tests were used to determine if warm season period or intake masses (DV) were different between categories of location and oiling (IV). Linear regression was used to determine if there was correlation between warm season period and intake masses. Fisher’s exact test was used to compare survival (DV) between categories of diagnosis (IV), either overall or within data subsets. All tests were two-tailed and used a significance level of 0.05.

Binary logistic regression was employed to determine which independent variables (IV) such as location, warm season period, oiling and intake mass had a significant effect in determining diagnosis (DV). The order of inclusion of variables in the logistic model and best model selection was based on the *P*-value of the Slope-equal-to-zero test (only variables with *P* > 0.1 were included) and Pearson’s Goodness-of-fit test.

All procedures in this study were approved by the Ethics Committee on Animal Use of the School of Veterinary Medicine and Animal Science of the University of São Paulo (CEUA 601415) and were authorized by Brazilian authorities (SISBIO 20825-6).

## Results

### Epidemiology of *Plasmodium* in penguins at rehabilitation centers

**Figure 2:**
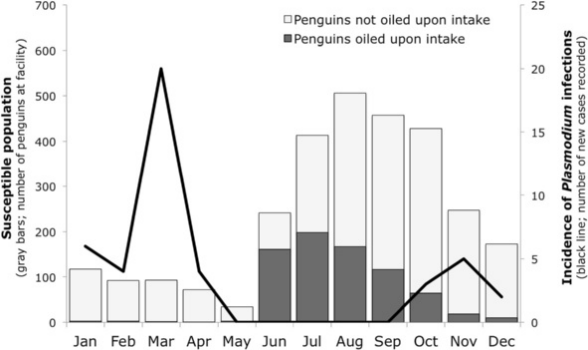
**Monthly distribution of** ***Plasmodium*** **infections in Magellanic penguins at rehabilitation facilities in Brazil.** Line represents the incidence of *Plasmodium* infections (number of cases first recorded at each month; right vertical axis). Bars represent the susceptible population (number of penguins that spent one or more days at the rehabilitation facilities in a given month; left vertical axis). Data is combined for all facilities and years.

Forty-four of the 774 MPs (5.68%) undergoing rehabilitation at facilities in six states along the coast of Brazil were identified as positive to *Plasmodium* (Figure 1 and Table 1). Positive individuals were identified at all states except São Paulo, and no individuals were positive upon intake. In all positive cases, clinical history and diagnostic results were consistent with the hypothesis that infection occurred during the stay at rehabilitation facilities. Details on individual rehabilitation history and clinical parameters of *Plasmodium*-positive individuals are provided in Additional file 2. A substantial proportion of positive cases (39%: 17/44) were concentrated in a single outbreak that occurred at Santa Catarina in March-April 2009. All avian malaria cases were first identified as positive between the months October and April, inclusive (Figure 2).

Individuals tested with PCR or with a combination of PCR and TBS were more frequently determined to be positive (7.50% and 6.32%, respectively) than those tested with TBS alone (1.79%) (*P* = 0.022). All samples identified as positive by TBS were positive in the corresponding PCR test, whereas only 45.5% (10/22) PCR-positive samples were identified as positive in the corresponding TBS.

When only individuals sampled systematically and tested with PCR (whether in combination with TBS or not) are considered, apparent prevalence was 7.75% (30/387) (Table 2). Based on this result, true prevalence was estimated between 6.6% and 13.5%. Logistic regression of this data subset (Log-likelihood = −43.029, *P* < 0.001; Pearson Goodness-of-fit test: χ^2^ = 145.68, df = 321, *P* > 0.99) revealed the following variables were significant determinants of positivity to *Plasmodium*: location (Santa Catarina in relation to Rio Grande do Sul: Z = 1.75, *P* = 0.080, b = 1.437, b_CI95%_ = −0.172 – 3.048, OR = 4.21, OR_CI95%_ = 0.84 – 21.06), warm season period (Z = 4.97, *P* < 0.001, b = 0.030, b_CI95%_ = = 0.018 – 0.042, OR = 1.03, OR_CI95%_ = 1.02 – 1.04) and intake mass (Z = 2.47, *P* = 0.013, b = 0.0014, b_CI95%_ = = 0.0003 – 0.0025, OR = 1.01, OR_CI95%_ = 1.01 – 1.01). Oiling did not have a significant effect (not oiled in relation to oiled; Z = 0.59, *P* = 0.554, b = 0.47, b_CI95%_ = = −1.10 – 2.05, OR = 1.61, OR_CI95%_ = 0.33 – 7.75).

**Table 2:** **Details of the diagnostic results in relation to sample collection and testing strategy**, **age group**, **oiling and survival**

**Sampling and screening**	**Age group**	**Oiling**	**Died**		**Survived**		**Total**
			**Positive**	**Negative**	**Positive**	**Negative**	
Systematically sampled	Juvenile	Oiled	3	92	0	81	176
and PCR-tested individuals		Not oiled	13	62	9	67	151
	Adult	Oiled	0	5	0	37	42
		Not oiled	4	6	1	7	18
Opportunistically sampled	Juvenile	Oiled	1	2	0	9	12
and/or non-PCR-tested individuals		Not oiled	7	129	5	213	354
	Adult	Oiled	1	5	0	11	17
		Not oiled	0	0	0	4	4
**Total**			29	301	15	429	774

Strong association/correlation was present amongst variables: between location and oiling (*P* < 0.001), warm season period (*P* < 0.001), and intake mass (*P* = 0.029); between oiling and warm season period (*P* < 0.001) and intake mass (*P* = 0.005); and between warm season period and intake mass (*P* = 0.001, R^2^ = 0.039, b > 0). Survival was significantly different between *Plasmodium*-positive and *Plasmodium*-negative individuals, with 66.6% of the *Plasmodium*-positive MPs dying during rehabilitation whereas just 46.21% of the *Plasmodium*-negative died (*P* = 0.037).

### *Plasmodium* lineages infecting penguins

Morphological characterization of parasites revealed the occurrence of at least four different morphospecies in 16 blood smear-positive individuals: *P*. (*Novyella*) *nucleophilum*, *P*. (*Haemamoeba*) *cathemerium*, *P*. (*Haemamoeba*) *tejerai* and *P*. (*Huffia*) *elongatum* (photomicrographs provided in Additional file 3 and in references [8] and [14]). In the blood smears of *P. cathemerium*, it should be noted that in addition to the well-defined elongated rod-shaped pigment granules with pointed ends that are unique to this species, unusual morphological characteristics were also noted: meronts were relatively small with scanty cytoplasm and young macrogametocytes frequently presented relatively large vacuoles surrounded by small round pigment granules.

**Figure 3:**
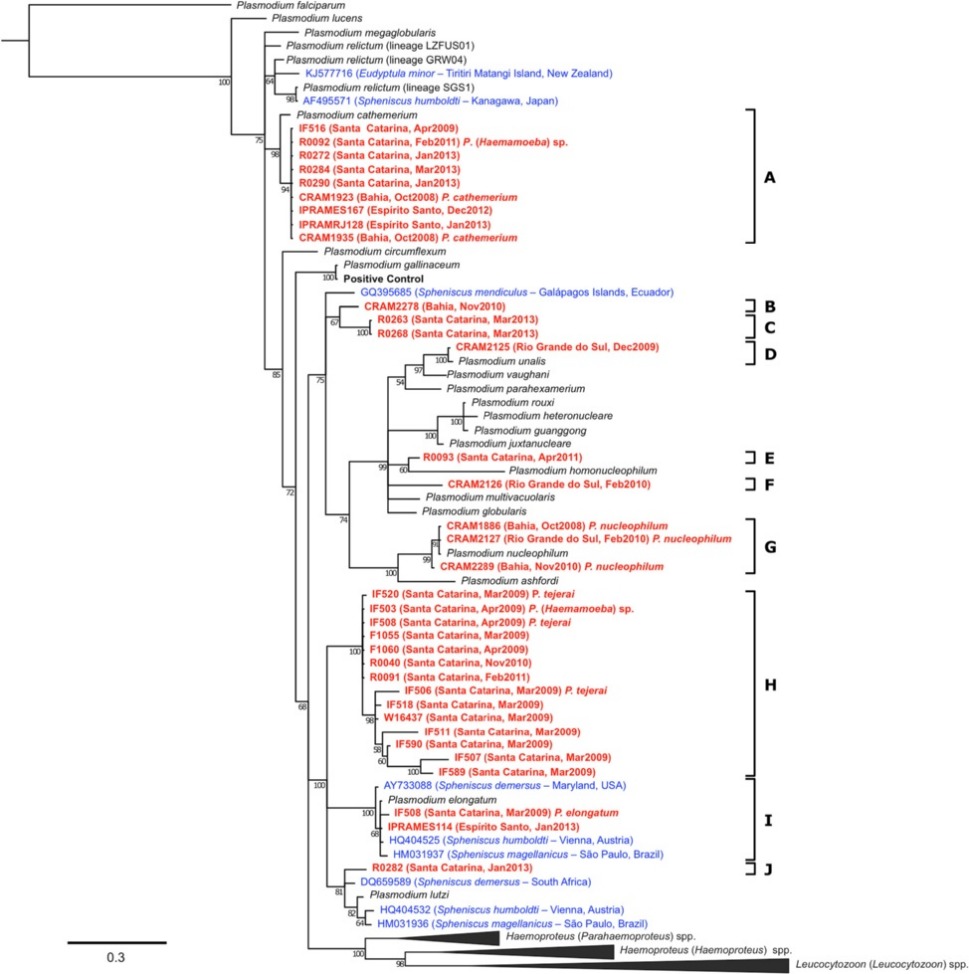
**Phylogenetic tree of the** ***Plasmodium*** **spp. lineages identified in penguins.** (red) Magellanic penguins undergoing rehabilitation along the coast of Brazil (this study), (blue) published penguin-infecting lineages, (black) reference lineages. Branch lengths are drawn proportionally to the extent of changes (scale bar is shown).

*Cyt*-*b* sequences were obtained from 34 of the 36 PCR-positive individuals (see Additional file 1), and phylogenetic analysis revealed that these lineages can be classified in 10 distinct clusters (Figure 3). Four clusters were confirmed as morphospecies based on parasite morphology in blood smears: *P. cathemerium* (cluster A), *P. nucleophilum* (cluster G), *P. tejerai* (cluster H), and *P. elongatum* (cluster I). Additionally, even though the morphology of *Plasmodium* lineage D (obtained from penguin CRAM2125) could not be observed in blood smears, the sequences from this lineage neatly clustered with a reference *P*. (*Novyella*) *unalis* lineage, with high probability (100) and sequence identity (444/445 nucleotides = 99.76%). Five phylogenetic lineages (B, C, E, F and J) did not cluster with any reference lineages nor with lineages previously obtained from penguins.

### Pathology of avian malaria in penguins

**Figure 4:**
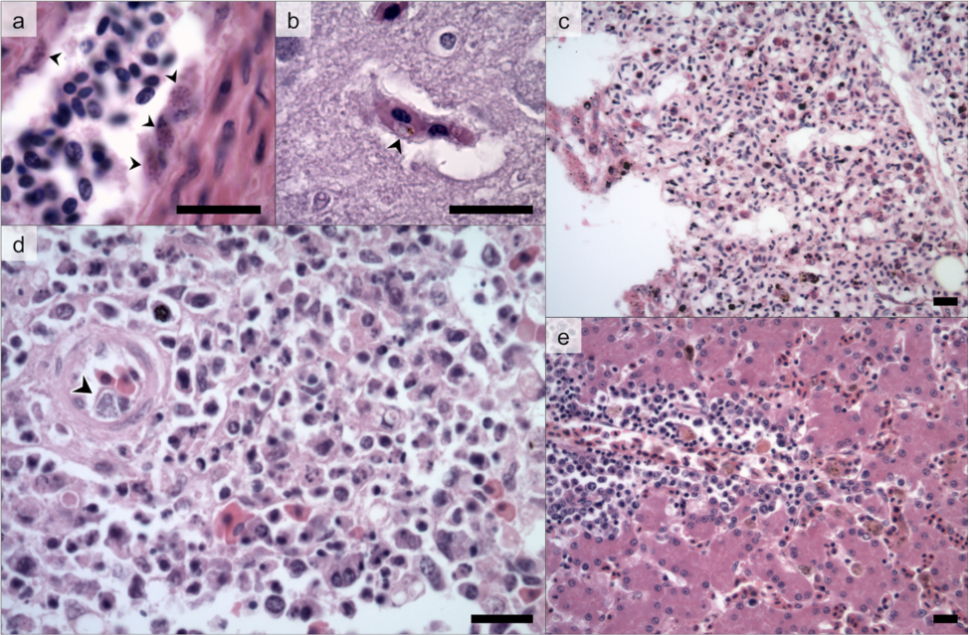
**Histological findings associated with avian malaria in Magellanic penguins.** **(a)** exoerythrocytic meronts in endothelial cells (arrowheads) within a liver arteriole (R0040, *P. tejerai*); **(b)** parasitized erythrocyte (arrowhead) within a cerebral blood vessel (CRAM2127, *P. nucleophilum*); **(c)** diffuse granulocytic interstitial pneumonia, congestion and edema (IF584, *P. tejerai*); **(d)** diffuse necrotizing splenitis with an exoerythrocytic meront within an endothelial cell of a central arteriole (arrowhead) (R0290, *P. cathemerium*); **(e)** multifocal perivascular mononuclear hepatitis, congestion and hemosiderosis (R0093, *Plasmodium* sp lineage E). Hematoxilin-Eosin. Scale bars = 15 μm.

Twenty-two *Plasmodium*-positive cases were examined by histopathology, and exoerythrocytic meronts were observed in 19 cases (86.4%). Meronts were present in macrophages and endothelial cells (Figure 4A), and occurred in a broad variety of tissues, especially in the heart, liver, lungs, spleen and kidneys.

*P. tejerai* was lethal to 75% of penguins infected (12/16), and tissue meronts were observed in all six *P. tejerai* cases examined by histopathology. *P. cathemerium* was identified in 9 MPs, three of which died (33% lethality); another two were euthanized for other reasons. Only one of three *P. nucleophilum*-infected MPs died, and necropsy revealed there were no tissue meronts but high numbers of intraerythrocytic parasites were present within blood vessels (Figure 4B); the concurrence of other significant pathological processes (severe splenic amyloidosis, helminthes within lungs and liver parenchyma and intestinal blood vessels) did not allow for a conclusion as to whether or not avian malaria was the cause of death. *P. elongatum* was identified in one dead MP, which did present tissue meronts. *P. unalis* was identified in one dead penguin, but no tissue meronts were observed and severe respiratory lesions indicated that aspergillosis was the primary cause of death. *Plasmodium* sp lineages E and J were each identified in one individual, and tissue meronts were present in each case. *Plasmodium* lineage C was identified in two euthanized individuals; tissue meronts were present in both cases but concurred with other significant lesions (severe aspergillosis accompanied by necrotizing pancarditis; amyloidosis and helminthes within air sacs).

Only one case of mixed infection resulted in death (*P. elongatum* + *P. tejerai*), however histopathology was not conducted and therefore the role of avian malaria towards the cause of death could not be determined. Diffuse interstitial pneumonia occurred in all examined cases (*n* = 22) and was most frequently granulocytic (82%) (Figure 4C). Multifocal hepatitis occurred in all examined cases (*n* = 20) as was most frequently mononuclear (70%) (Figure 4D); hepatic necrosis and ductal hyperplasia were present in 20% and 25% of cases, respectively. Splenitis occurred in 75% of cases (*n* = 20) and was most often granulocytic (67%); necrotizing splenitis and/or lymphocytolysis was observed in 65% of cases (Figure 4E), and massive splenic hemorrhages were observed in two cases (10%). Hematopoiesis (60%) and hemosiderosis (90%) were frequently observed in the spleen and/or liver (*n* = 20). Myocarditis was observed in 33.3% of cases (*n* = 21) and was most frequently granulocytic (57%).

## Discussion

Avian malaria has been widely recognised as one of the most significant infectious diseases for wild and captive penguins [3,4,27]. Our findings demonstrate that *Plasmodium* spp. infect Magellanic penguins at several rehabilitation centers along the Brazilian coast, leading to substantial levels of mortality, and limiting the success of rehabilitation efforts for this species at these centers.

### Epidemiology of avian malaria in penguins at rehabilitation centers

While the overall apparent prevalence ranged from 2.1% to 13.8% among rehabilitation centers, when only samples collected and tested in a systematic manner with highly sensitive diagnostic methods were considered, the apparent prevalence was 7.8%. With 95% confidence, true prevalence was estimated between 6.6% and 13.5%. The only comparable data in the literature are provided by the Southern African Foundation for the Conservation of Coastal Birds (SANCCOB), an organization that rehabilitates African penguins (*Spheniscus demersus*) in South Africa; 17-34% of the African penguins admitted by SANCCOB in 2001–2002 were blood smear-positive to *Plasmodium* at some point during their rehabilitation [27].

We found that 5% of MPs that survived through rehabilitation were *Plasmodium*-positive at some point, compared to 10.8% of the deceased MPs, with a 44% higher mortality in *Plasmodium*-positive penguins compared to *Plasmodium*-negative (66.6% vs. 46.2%). These results contrast with the observed epidemiology at SANCCOB, where the proportion of animals released of *Plasmodium*-positive individuals is not significantly different from that of the overall rehabilitation population [27].

There is, however, a critical difference to be considered when comparing our findings with those of SANCCOB: there are no records of *Plasmodium* infections in wild MPs, whereas African penguins are infected in the wild [5]. Therefore, whilst we found no evidence to indicate that the MPs we studied carried the infection from the wild, at SANCCOB 30-35% of the *Plasmodium*-positive African penguins are already positive upon intake [27]. In this context, the epidemiology of avian malaria in Magellanic penguins in rehabilitation centers may resemble more that of captive penguins in the Northern Hemisphere than that of their African counterparts.

In the Northern Hemisphere, it is well established that the occurrence of avian malaria in penguins is strongly modulated by seasonality, with cases concentrated in the Boreal spring-summer due to climate-mediated fluctuations in mosquito abundance [10,28,29]. We observed a consistent concentration of all *Plasmodium*-positive cases in the Austral spring-summer (October to April), with the probability of infection being positively correlated with the total number of days spent in rehabilitation during that time of the year. In contrast, most MPs are received for rehabilitation along the Brazilian coast during winter months (June to September) (Figure 2), which relates to the species’ wintering ecology [9]. Current rehabilitation protocols require the release of MPs is to be avoided during the summer months in recognition of the species’ migration [15]. As a result, individuals that were unable to meet the release criteria until spring are retained for release in the subsequent winter; a lower number of individuals are also admitted for rehabilitation during summer.

Surprisingly, body mass upon intake was found to be a positively-correlated predictor of *Plasmodium* infection, indicating that MPs admitted with higher body mass have a higher probability of becoming infected with *Plasmodium*. A possible explanation is that MPs admitted during summer months are often admitted due to moult problems and as a result may have relatively higher body mass (RETV, personal observations).

The rehabilitation facility studied in Santa Catarina had a substantially higher *Plasmodium* incidence than the other facilities involved in this study. The facility is located within a State Park (Parque Estadual do Rio Vermelho) with penguin enclosures directly under tree cover of Atlantic forest and less than 10 metres away from a large freshwater lake (Lagoa da Conceição). This provides an ideal environment for mosquito proliferation and close proximity to an abundant and diverse avifauna. Additionally, this facility rehabilitates not only marine animals but also terrestrial wildlife, including birds apprehended from illegal trade. The higher apparent prevalence of MPs at this facility may reflect not a regional difference in susceptibility or lineage virulence, but is more likely to be the consequence of an increased frequency of inoculation due to close contact with mosquitoes and other birds acting as proximal reservoirs of infection.

### *Plasmodium* lineages infecting penguins and their pathology

It has been generally accepted that *P. relictum* and *P. elongatum* are the two most significant species of *Plasmodium* responsible for avian malaria in penguins [3,5,6], in addition to an isolated record of *P. juxtanucleare* [7]. In recent studies [8,14] we have documented the role of *P. tejerai* in causing an avian malaria outbreak in MPs at Santa Catarina.

Our results demonstrate that a broad variety of *Plasmodium* species can be found in penguins undergoing rehabilitation in South America, including three species that had not yet been demonstrated to infect penguins (*P. cathemerium*, *P. nucleophilum* and *P. unalis*) and 5 lineages that albeit unidentified clearly correspond to *Plasmodium* species that also have not yet been reported in penguins (lineages B, C, E, F and J). Such diversity of plasmodia corroborates the interpretation that the infection of captive penguins results from local mosquitoes inoculating penguins with *Plasmodium* spp. acquired from other birds surrounding of the penguin exhibits [12,29-31].

It is worth noting that *P. cathemerium*, *P. elongatum*, *P. nucleophilum* and *P. relictum* are renowned as generalist parasites with low host-specificity, infecting a broad range of avian species in several taxonomic orders [32]. In this sense, our findings suggest that the predominance of *P. relictum* in Europe and Asia and *P. relictum* and *P. elongatum* in North America might not necessarily indicate a particular susceptibility of penguins to those parasites, but perhaps reflects their natural abundance in those regions.

Although we observed mature *P. cathemerium* microgametocytes with well-defined elongated rod-shaped pigment granules with pointed ends, which are the defining features of *P*. (*Haemamoeba*) *cathemerium* [16], in all cases we also observed late trophozoites and young macrogametocytes with relatively large vacuoles surrounded by small round pigment granules and relatively small meronts with scanty cytoplasm (Additional file 3), which are uncharacteristic to *P. cathemerium*. These findings may be interpreted as: (a) a host-specific morphological variation of *P. cathemerium*, (b) a variant or subspecies of *P. cathemerium*, (c) co-infection with a secondary unidentified lineage, or (d) a novel and yet undescribed *Plasmodium* species whose morphological characteristics overlap with those of *P. cathemerium*. Considering the molecular evidence indicating a consistently high sequence identity and phylogenetic proximity with a reference lineage of *P. cathemerium*, along with a failure to retrieve sequences of another *Plasmodium* lineage in the same samples, we believe there is sufficient evidence to identify these lineages as *P. cathemerium* and that hypothesis (a) or (b) are most likely.

### Pathogenicity of *Plasmodium* to penguins

Even though penguins seem to be susceptible to infection by a variety of *Plasmodium* lineages occurring regionally, we cannot say that all lineages have similar epidemiology or pathogenicity. In this study we found that *P. tejerai* and *P. cathemerium* were lethal (75% and 33% of MPs in which they were detected, respectively) whilst other lineages had only a few cases recorded and/or could not be demonstrated as the leading factor causing death. These findings, combined with previous reports that albeit less frequent *P. relictum* tends to produce more severe disease than *P. elongatum* in penguins [29,31], raises the question on whether the *Plasmodium* subgenus *Haemamoeba* is more pathogenic to these birds than other subgenera of *Plasmodium*. Comparative pathology through experimental inoculation in domestic birds may assist in clarifying whether pathogenicity is intrinsically higher for these lineages or if it reflects a susceptibility bias present in penguins.

Overall, however, the histopathological lesions observed in this study were generally consistent among lineages. The most prominent pathological processes were granulocytic pneumonia and splenitis and mononuclear hepatitis; these were probably the effect of vasculitis associated with the proliferation of *Plasmodium* within endothelial cells of these tissues. In most cases, death likely culminated as a result of respiratory insufficiency from the marked pneumonia, congestion and edema. These lesions are not unlike those reported in *P. relictum* and *P. elongatum* infections in penguins in zoos and aquaria in the Northern Hemisphere [10,11,33].

In contrast, no exoerythrocytic meronts were observed in the tissues of the only MP that died with a *P. nucleophilum* infection, whereas a high number of intraerythrocytic parasites was observed within blood vessels. Similarly, the only individual infected with *P. unalis* did not present detectable exoerythrocytic meronts. This distinct pattern may indicate a different stage of infection or a distinct pathogenesis. Future studies will be welcome to clarify whether this is a consistent pattern in infections by these lineages in penguins and which pathophysiological mechanisms are involved.

### Concurrent diseases

Two of the *Plasmodium*-positive MPs identified in this study had also been identified as positive to *Avipoxvirus* in a previous study [34]. This is probably not uncommon as both pathogens are mosquito-borne, however it may confuse interpretation of pathological findings. In particular, the only two individuals studied by Niemeyer et al. [34] that presented necrotizing splenitis were found to be *Plasmodium*-positive in this study. This might indicate that such severe lesions were related to avian malaria and not poxvirosis, and that *Avipoxvirus* might not have been as pathogenic as originally thought.

Other concurrent diseases included aspergillosis, gastrointestinal helminthiasis, spleen amyloidosis, cholestasis, unidentified myocardium cysts, and helminthes in the lungs, liver, air sacs and skin (Additional file 3). Some of these findings, such as aspergillosis and gastrointestinal helminthiasis have been previously reported in penguins with avian malaria [10,35]. However, there have been no reports of helminthes in the respiratory system, skin or air sacs of MPs [3]. Furthermore, the myocardium cysts herein observed clearly were not *Plasmodium* and could correspond to either protozoan or metazoan parasites. Additional studies will be conducted to clarify the identity and significance of these parasites.

### Implications for rehabilitation and conservation

The prevention of avian malaria in penguins in Northern Hemisphere zoos has largely relied on the oral administration of primaquine during summer [15]. In Brazil, primaquine commerce is restricted by the government due to concerns of potential resistance in human malaria, and therefore this drug cannot be acquired or used by rehabilitation centers. As a result, the centers are forced to employ other prevention strategies, namely the physical isolation of penguins from mosquitoes, which is often challenging and costly.

Our study sheds light on a positive aspect of the epidemiology of this disease at rehabilitation centers, namely that the periods in which *Plasmodium* infections occur (summer) is directly opposite to the period in which there are the most penguins in rehabilitation (winter). Consequently, one of the key strategies for the prevention of avian malaria in these facilities might be to develop rehabilitation protocols that allow the shortening of the time needed by the penguins to achieve the fit-to-release criteria, so that they can be released before the summer. As a result, these facilities would benefit from narrowing their malaria-prevention efforts to a relatively lower number of individuals (those received and/or maintained between October and April), becoming more effective in the prevention and early diagnosis. This is a relevant implication not only for permanent rehabilitation efforts, but also for oil spill responses involving penguins, when the physical and human resources required for malaria prevention, diagnosis and treatment may be substantial [35] and potentially beyond the capacity of those involved.

A number of MPs herein examined were released despite having been *Plasmodium*-positive at some point. These individuals were clinically healthy and passed the standard release criteria [15] and were blood smear-negative or, in a few cases, blood samples were collected but not examined in time before release. In the cases where blood smears were negative, it must be considered that even non-parasitemic penguins can relapse if treated with corticosteroids – and presumably the same would occur if they became stressed – due to the persistence of exoerythrocytic meronts [30]. In the cases where samples could not be tested before release, this exposes a potential dillema that is common in oil spill responses, where it is not always feasible to test the large numbers of individuals in a brief period [36,37].

Brossy et al. [38] expressed concern on the potential of rehabilitation centers releasing African penguins with blood parasites, and perhaps this concern should be even greater for MPs considering that *Plasmodium* has yet to be recorded in this species in the wild. In the case of MPs, however, because climate and environmental conditions are generally adverse and mosquitoes occur very scarcely in the southeastern coast of Argentina [39,40] and are absent at the Malvinas-Falkland Islands [41], the probability of *Plasmodium* transmission from a rehabilitated penguin to a wild penguin in these regions is very low. Even so, it is important to emphasize that pathogen spill-over to wild populations should remain a prime and critical concern for rehabilitation centers, and that even a low non-zero probability is nonetheless a significant risk to be considered and addressed.

Rehabilitation facilities on the Pacific coast of South America may be in a different situation. There are reports of avian malaria in MPs undergoing rehabilitation in Chile [13], and even though no studies have detected blood parasites in penguins in Chile [5,6], both ecological models [40] and blood parasite studies in other avian species [42] consistently indicate that mosquitoes are abundant on the Southwestern coast of South America. Studies examining the occurrence of blood parasites in the Chilean populations of MPs are therefore urgently required, and rehabilitation facilities in the region should remain cautious of potentially releasing *Plasmodium*-positive individuals back into the wild.

## Additional files

Additional file 1:
**Public database ascension numbers.** Genbank and MalAvi ascension numbers for the sequences obtained or included in the analyses. Taxonomic names within brackets indicate the taxon to which the species is presumed to correspond on the basis of phylogenetic analyses of the cytochrome b mitochondrial gene. Additional file 2:
**Individual details of** ***Plasmodium*** **-positive Magellanic penguins.** Spreadsheet with rehabilitation history and clinical parameters of *Plasmodium*-positive Magellanic penguins identified in this study. Additional file 3:
***Plasmodium*** **spp. in Giemsa-stained blood smears of Magellanic penguins.** Photomicrographs: *P. nucleophilum* (CRAM2127): (a,b) trophozoites, (c,d) meronts, (e) coinfection by erythrocytic meront and microgametocyte, (f) macrogametocyte, (g) microgametocyte, (h) co-infection by macro and microgametocyte; *P. cathemerium* (CRAM1923): (i) trophozoite, (j,k) meronts, (l-n) macrogametocytes, (o,p) microgametocytes. Scale bar = 5 μm.

## Abbreviations

DV: Dependent variable
HP: Histopathology
IV: Independent variable
MPs: Magellanic penguins
PCR: nested polymerase chain reaction
SANCCOB: Southern African Foundation for the Conservation of Coastal Birds
TBS: Thin blood smears
