# Supplementary material for: Epidemiology and pathology of avian malaria in penguins undergoing rehabilitation in Brazil
Source: Vet Res. 2015 Mar 13;46:30. doi: 10.1186/s13567-015-0160-9 (PMC4357068; doi:10.1186/s13567-015-0160-9)
Supplement: Additional file 1: — Public database ascension numbers. Genbank and MalAvi ascension numbers for the sequences obtained or included in the analyses. Taxonomic names within brackets indicate the taxon to which the species is presumed to correspond on the basis of phylogenetic analyses of the cytochrome b mitochondrial gene. [file 13567_2015_160_MOESM1_ESM.docx]

| Sample ID / Reference | Species or lineage | Ascension number |
| --- | --- | --- |
| CRAM1886 | *P. nucleophilum* | KM365040 |
| CRAM1923 | *P. cathemerium* | KJ577720 |
| CRAM1935 | *P. cathemerium* | KM365041 |
| CRAM2125 | [*P. unalis*] | KJ577721 |
| CRAM2126 | [*Plasmodium* sp lineage F] | KJ577722 |
| CRAM2127 | *P. nucleophilum* | KJ577723 |
| CRAM2278 | [*Plasmodium* sp lineage B] | KJ577724 |
| CRAM2289 | *P. nucleophilum* | KJ577725 |
| F1055 | [*P. tejerai*] | KJ577726 |
| F1060 | [*P. tejerai*] | KJ577727 |
| IF503 | *P*. (*Haemamoeba*) sp [*P. tejerai*] | KJ575551 |
| IF506 | *P. tejerai* | JX272844 |
| IF507 | [*P. tejerai*] | KJ575544 |
| IF508A | *P. elongatum* | HQ591360 |
| IF508B | *P. elongatum* | KJ575552 |
| IF511 | [*P. tejerai*] | KJ575545 |
| IF516 | [*P. cathemerium*] | KJ575553 |
| IF518 | [*P. tejerai*] | KJ575547 |
| IF520 | *P. tejerai* | HQ591361 |
| IF589 | [*P. tejerai*] | KJ575548 |
| IF590 | [*P. tejerai*] | KJ575549 |
| IPRAM-ES114 | [*P. elongatum*] | KJ577717 |
| IPRAM-ES167 | [*P. cathemerium*] | KJ577718 |
| IPRAM-RJ128 | [*P. cathemerium*] | KJ577719 |
| R0040 | [*P. tejerai*] | KJ577728 |
| R0091 | [*P. tejerai*] | KJ577729 |
| R0092 | *P*. (*Haemamoeba*) sp [*P. cathemerium*] | KJ577730 |
| R0093 | [*Plasmodium* sp lineage E] | KJ577731 |
| R0263 | [*Plasmodium* sp lineage C] | KJ577732 |
| R0268 | [*Plasmodium* sp lineage C] | KJ577733 |
| R0272 | [*P. cathemerium*] | KJ577734 |
| R0282 | [*Plasmodium* sp lineage J] | KJ577735 |
| R0284 | [*P. cathemerium*] | KJ577736 |
| R0290 | [*P. cathemerium*] | KJ577737 |
| W16437 | [*P. tejerai*] | KJ575550 |
| Suplick et al., 1988 | *P. falciparum* | M99416 |
| Beadell & Fleischer, 2005 | *Plasmodium* sp [*P. elongatum*] | AY733088 |
| Beadell et al., 2006 | [*Plasmodium* sp] | DQ659589 |
| Ejiri et al., 2009 | [*Plasmodium* sp] | AF495571 |
| Levin et al., 2009 | [*Plasmodium* sp] | GQ395685 |
| Levin et al., 2009 | [*H*. (*Parahaemoproteus*) sp] | GQ395686 |
| Bueno et al., 2010 | *Plasmodium* sp [*P*. (*Haemamoeba*) sp] | HM031936 |
| Bueno et al., 2010 | *Plasmodium* sp [*P. elongatum*] | HM031937 |
| Van Rensburg, 2010 | P. (*Haemamoeba*) sp [*P. relictum*] | KJ577716 |
| Dinohpl et al., 2011 | [*P*. (*Haemamoeba*) sp] | HQ404532 |
| Dinohpl et al., 2011 | *Plasmodium* sp [*P. elongatum*] | HQ404525 |
| Argilla et al., 2013 | *Leucocytozoon* sp | JX569269 |
| Argilla et al., 2013 | *Leucocytozoon* sp | JX569270 |
| Cannell et al., 2013 | [*H*. (*Parahaemoproteus*) sp] | KC121053 |
| MalAvi | *P. ashfordi* | GRW02 |
| MalAvi | *P. cathemerium* | SEIAUR01 |
| MalAvi | *P. circumflexum* | TURDUS1 |
| MalAvi | *P. elongatum* | GRW06 |
| MalAvi | *P. gallinaceum* | GALLUS01 |
| MalAvi | *P. globularis* | ANLAT01 |
| MalAvi | *P. guanggong* | PYCJOC01 |
| MalAvi | *P. heteronucleare* | UNKNOWN01 |
| MalAvi | *P. homonucleophilum* | SW2 |
| MalAvi | *P. juxtanucleare* | GALLUS02 |
| MalAvi | *P. lutzi* | TFUS05 |
| MalAvi | *P. lucens* | CYAOLI09 |
| MalAvi | *P. megaglobularis* | PYSUN1 |
| MalAvi | *P. multivacuolaris* | ANLAT07 |
| MalAvi | *P. nucleophilum* | DENPET03 |
| MalAvi | *P. parahexamerium* | ALEDIA02 |
| MalAvi | *P. relictum* | GRW04 |
| MalAvi | *P. relictum* | LZFUS01 |
| MalAvi | *P. relictum* | SGS1 |
| MalAvi | *P. rouxi* | UNKNOWN02 |
| MalAvi | *P. unalis* | TFUS06 |
| MalAvi | *P. vaughani* | SYAT05 |
| MalAvi | *H. attenuatus* | ROBIN1 |
| MalAvi | *H. balmorali* | SFC1 |
| MalAvi | *H. belopolskyi* | HIICT1 |
| MalAvi | *H. columbae* | HAECOL1 |
| MalAvi | *H. concavocentralis* | HAWF2 |
| MalAvi | *H. cyanomitrae* | CYAOLI03 |
| MalAvi | *H. enucleator* | ALCLEU01 |
| MalAvi | *H. homobelopolskyi* | PLOMEL02 |
| MalAvi | *H. homovelans* | PICAN02 |
| MalAvi | *H. iwa* | REMIN01 |
| MalAvi | *H. jenniae* | CREFUR01 |
| MalAvi | *H. lanii* | RB1 |
| MalAvi | *H. magnus* | ROFI1 |
| MalAvi | *H. majoris* | CCF5 |
| MalAvi | *H. motacillae* | YWT1 |
| MalAvi | *H. micronuclearis* | VILWE1 |
| MalAvi | *H. minutus* | TURDUS2 |
| MalAvi | *H. multipigmentatus* | COLTAL01 |
| MalAvi | *H. nucleocondensus* | GRW01 |
| MalAvi | *H. nucleofascialis* | MALRUB02 |
| MalAvi | *H. pallidulus* | SYAT03 |
| MalAvi | *H. pallidus* | COLL2 |
| MalAvi | *H. parabelopolskyi* | SYAT01 |
| MalAvi | *H. paranucleophilus* | MALRUB01 |
| MalAvi | *H. paruli* | TABI02 |
| MalAvi | *H. passeris* | PADOM05 |
| MalAvi | *H. payevskyi* | RW1 |
| MalAvi | *H. sacharovi* | MOD01 |
| MalAvi | *H. sanguinis* | BUL1 |
| MalAvi | *H. syrnii* | STAL2 |
| MalAvi | *H. tartakovskyi* | ALARV03 |
| MalAvi | *H. thraupi* | POILI01 |
| MalAvi | *H. vacuolatus* | ANLAT02 |
| MalAvi | *H. valkiunasi* | FREAND01 |
| MalAvi | *H. vieronis* | VIGIL01 |
| MalAvi | *L. buteonis* | BUTREG01 |
| MalAvi | *L. fringillinarum* | TFUS04 |
| MalAvi | *L. majoris* | CB1 |
| MalAvi | *L. mathisi* | ACCOP01 |
| MalAvi | *L. quynzae* | HEAME01 |
| MalAvi | *L. sabrazesi* | GALLUS08 |
| MalAvi | *L. schoutedeni* | GALLUS06 |
| MalAvi | *L. toddi* | ACCFRA01 |
| MalAvi | *L. ziemanni* | BUBO01 |
