# Supplementary figures and images for: Epidemiology and pathology of avian malaria in penguins undergoing rehabilitation in Brazil
Source: Vet Res. 2015 Mar 13;46:30. doi: 10.1186/s13567-015-0160-9 (PMC4357068; doi:10.1186/s13567-015-0160-9)

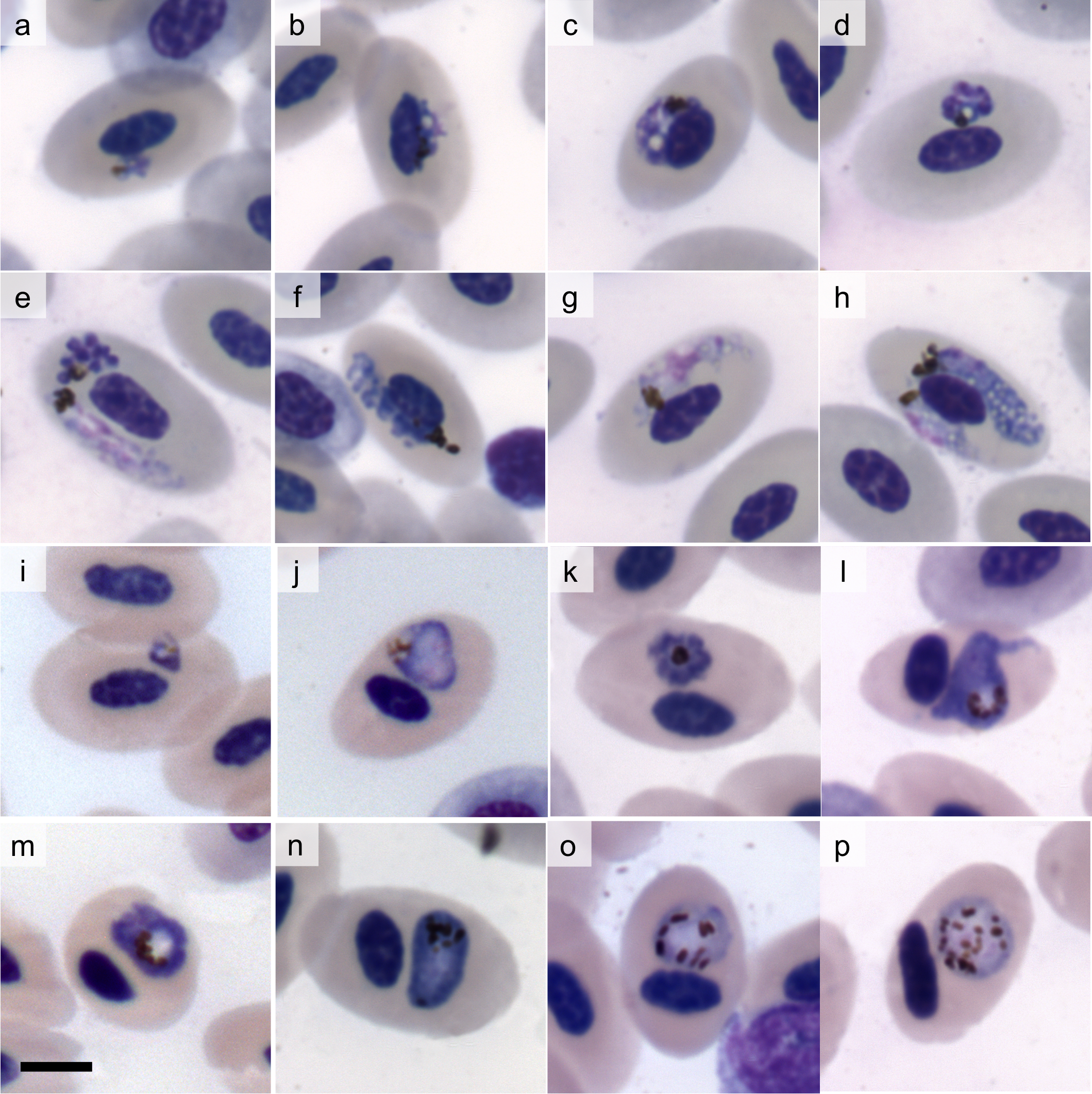

Supplement: Additional file 3: — Plasmodium spp. in Giemsa-stained blood smears of Magellanic penguins. Photomicrographs: P. nucleophilum (CRAM2127): (a,b) trophozoites, (c,d) meronts, (e) coinfection by erythrocytic meront and microgametocyte, (f) macrogametocyte, (g) microgametocyte, (h) co-infection by macro and microgametocyte; P. cathemerium (CRAM1923): (i) trophozoite, (j,k) meronts, (l-n) macrogametocytes, (o,p) microgametocytes. Scale bar = 5 μm. [file 13567_2015_160_MOESM3_ESM.png]
